# Vertical uniformity of cells and nuclei in epithelial monolayers

**DOI:** 10.1038/srep19689

**Published:** 2016-01-22

**Authors:** Srujana Neelam, Peter Robert Hayes, Qiao Zhang, Richard B. Dickinson, Tanmay P. Lele

**Affiliations:** 1 Department of Chemical Engineering, University of Florida, Gainesville, FL 32611; 2 J. Crayton Pruitt Family Department of Biomedical Engineering, University of Florida, Gainesville FL 32611 USA

## Abstract

Morphological variability in cytoskeletal organization, organelle position and cell boundaries is a common feature of cultured cells. Remarkable uniformity and reproducibility in structure can be accomplished by providing cells with defined geometric cues. Cells in tissues can also self-organize in the absence of directing extracellular cues; however the mechanical principles for such self-organization are not understood. We report that unlike horizontal shapes, the vertical shapes of the cell and nucleus in the z-dimension are uniform in cells in cultured monolayers compared to isolated cells. Apical surfaces of cells and their nuclei in monolayers were flat and heights were uniform. In contrast, isolated cells, or cells with disrupted cell-cell adhesions had nuclei with curved apical surfaces and variable heights. Isolated cells cultured within micron-sized square wells displayed flat cell and nuclear shapes similar to cells in monolayers. Local disruption of nuclear-cytoskeletal linkages resulted in spatial variation in vertical uniformity. These results suggest that competition between cell-cell pulling forces that expand and shorten the vertical cell cross-section, thereby widening and flattening the nucleus, and the resistance of the nucleus to further flattening results in uniform cell and nuclear cross-sections. Our results reveal the mechanical principles of self-organized vertical uniformity in cell monolayers.

Cellular cytoskeletal elements self-assemble into a diverse variety of structures that generate mechanical forces to establish cell and nuclear shape^1^,^2^,^3^, position intracellular organelles^4^, and traffic proteins and organelles to locations in the cell^3^. Recent efforts that cultured cells on micro-patterned extracellular matrix proteins have demonstrated that uniformity from cell to cell emerges in the spatial positioning of the centrosome, the Golgi apparatus and the nucleus^5^, the spatial assembly of actomyosin bundles and adhesions sites^5^, traction force patterns^6^,^7^, microtubule assembly^8^ and mitotic spindle orientation^9^. Culturing cells on micropatterned ECM islands allows the directional control of lamellipodial extensions^10^, and patterns of cell motility can emerge on micropatterned islands^11^. Recently, directed self-assembly of cytoskeletal structures has been demonstrated *in vitro* through the patterning of adhesive extracellular matrix proteins, and has helped understand the mechanisms by which uniformity of F-actin self-assembly may emerge inside cells^12^.

Epithelial cells in organs also have regular shapes and regular positioning of organelles like the nucleus and the centrosome, cytoskeletal structures, and membrane localization of specialized receptors that are important for their tissue-specific functions^13^. The mechanical principles that allow external control of assembly of intracellular structures may also enable the establishment of regular cell shape and structure in tissues^14^. For example, spatial variations in the mechanical properties of the extracellular matrix have been suggested to drive lung morphogenesis^15^. Cell shape control by spatially varying mechanical cues can also govern the process of angiogenesis^16^. While such evidence shows that directed self-assembly of cytoskeletal structures due to local variations in extracellular cues can participate in the dynamic development of complex tissues, cells can also self-assemble into uniform patterns and shapes in the absence of external cues. For example, breast epithelial cells self-organize into three-dimensional shapes with regular cell shapes and nuclear positions *in vitro*^17^ and *in vivo*^18^. However, the mechanical principles by which regular intracellular structure can emerge in tissues are not well-understood.

Here we imaged and reconstructed the three-dimensional shapes of cells and nuclei in epithelial cell monolayers. Despite the irregularity in cell shapes and nuclear shapes in the x-y plane, the heights of the apical surfaces of the cells and the nuclei were remarkably uniform in the z- dimension. This uniformity depended on intact cell-cell adhesions and an intact LINC complex. We explain the results with a simple model of competition between cell-cell pulling forces and nuclear resistance to further flattening.

## Results

### Vertical uniformity in epithelial monolayers

**Figure 1:**
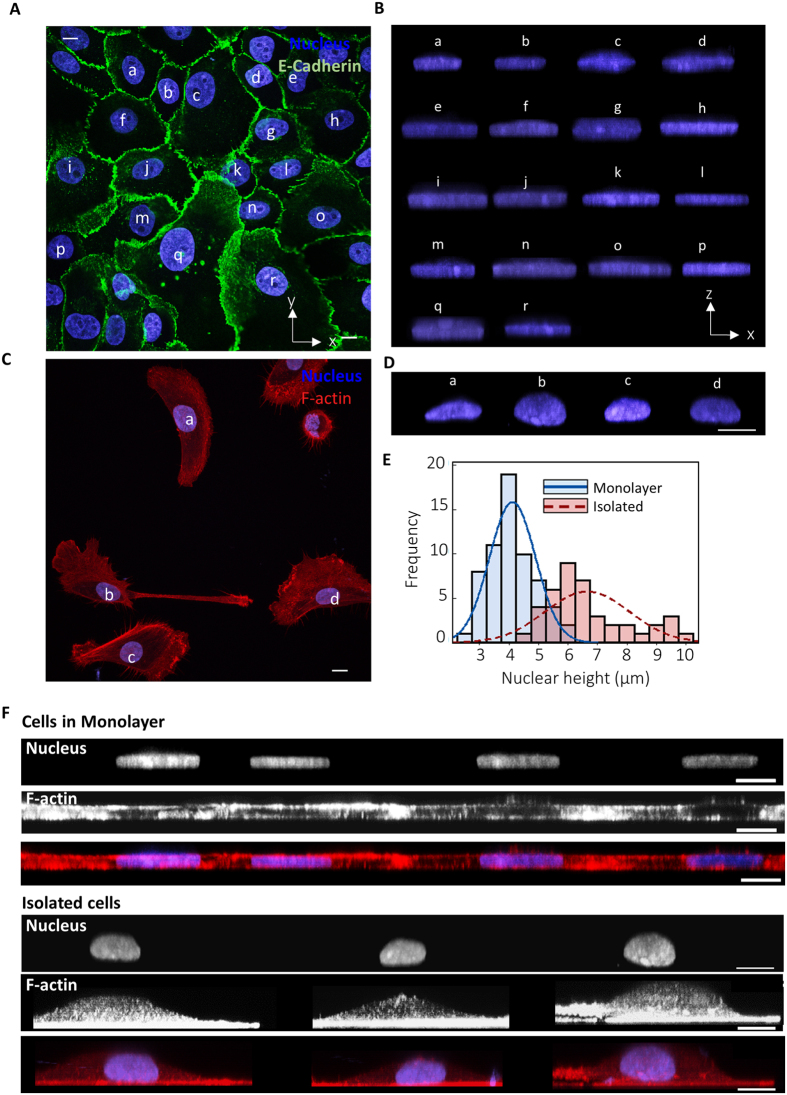
Uniform vertical shapes of nuclei and cells in breast epithelial monolayers. (**A**) Shows the x-y view of MCF 10A cells in a monolayer. Shown in (**B**) is the x-z view of the corresponding nuclei labelled in (**A**). Similarly (**C**) shows the x-y view of isolated MCF 10A cells and (**D**) shows the x-z view of the corresponding nuclei. (**E**) Histograms compare the frequency distribution of nuclear heights of cells in monolayers and in isolated cells. Variance in nuclear heights is higher in isolated cells than in monolayers (also confirmed by an F-test in Table 1), n = 63 for cells in monolayers and n = 42 for isolated cells (**F**) x-z views of nuclei, F-actin and overlays. Cell shapes in monolayers are flat with the nucleus tightly enclosed by the cell shape. Isolated cells do not have flat apical surfaces. Scale bar is 10 μm for all images.

We imaged cells and nuclei in MCF10A monolayers with confocal microscopy and developed x-z views of the nucleus (Fig. 1A,B). The x-z shapes of nuclei had remarkable uniformity. Nuclear height was nearly uniform, and the apical nuclear surface was nearly flat across cells separated by hundreds of microns in the monolayer (Fig. 1B), unlike the clearly variable shapes and curved nuclear apexes in isolated cells (Fig. 1C,D). Comparison of frequency distributions of nuclear height confirms the greater uniformity of nuclear heights in monolayers (also confirmed by an F-test comparing variances, Fig. 1E and Table 1). In contrast, x-y cross-sections were equally variable for cells in monolayers compared to isolated cells (Figure S1). We next examined the x-z shape of the cell by imaging F-actin distribution. Cells in monolayers had flat apical surfaces in close apposition to the nuclear apex, while in isolated cells, the cell apex was curved similar to the curved nuclear apex (Fig. 1F).

**Figure 2:**
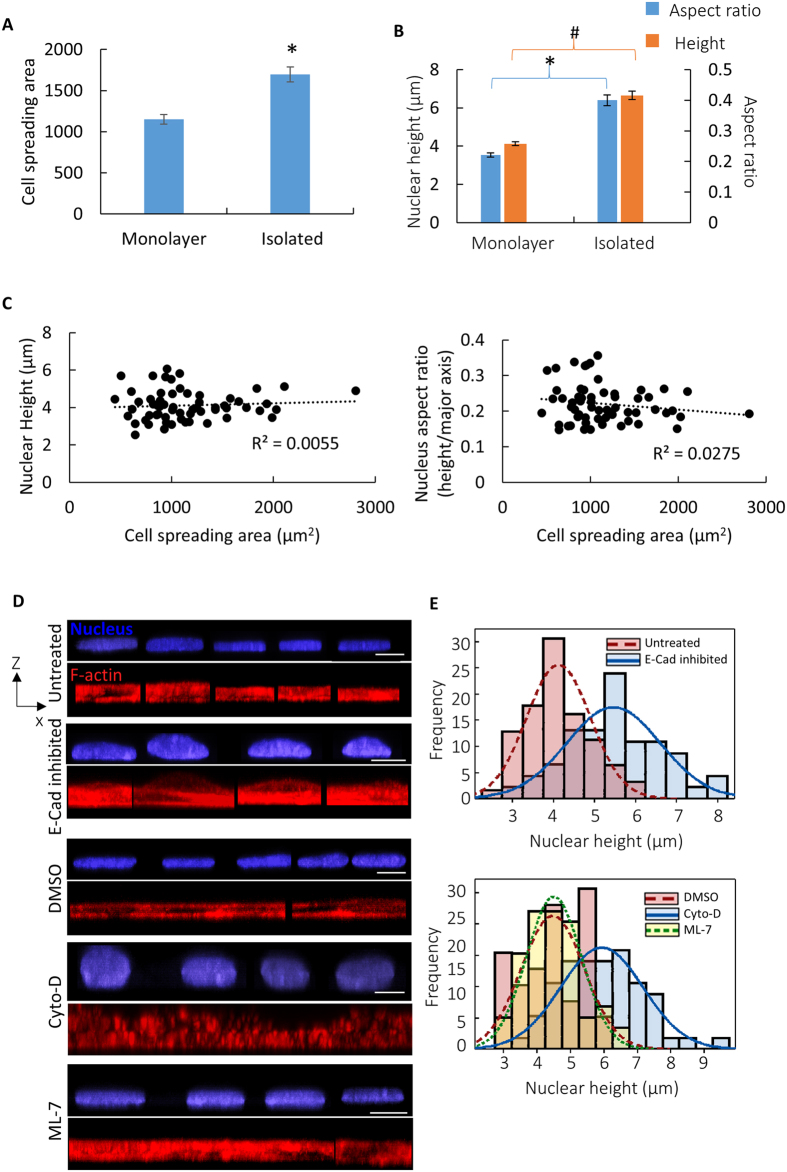
E-cadherin adhesions but not myosin activity is required to maintain nuclear uniformity. (**A**) Plot shows that the cells spread less in monolayers than in isolated cells.*P < 0.05. (**B**) Plot shows that the nucleus is flatter in the monolayer compared to isolated cells. *P < 0.05, ^*#*^*P* < 0.05, for all the indicated comparisons, n = 63 for cells in monolayers and n = 42 for isolated cells. Values are the mean ± SEM. (**C**) No correlation is observed between nuclear height or aspect ratio and the area of cell spreading in monolayers. (**D**) x-z views of cells and nuclei in monolayers, monolayers treated with Cytochalasin-D (Cyto-D), anti E-cadherin (E-cadherin inhibited) or ML-7. Nuclear shapes lose their uniform flat appearance in cytochalasin-D treated and E-cadherin inhibited monolayers. The nuclei in ML-7 treated monolayers showed no change in shape. F-actin stained cell shapes are seen to closely correlate with nuclear shape. Scale bar is 10 μm in all images. (**E**) Frequency distribution of the nuclear heights for control, DMSO control, Cyto-D treated, E-cadherin inhibited and ML-7 treated cells. Cyto-D treatment and E-cadherin inhibition increases variance in nuclear shapes (also see F-test in Table 1).

We have previously shown that nuclear height in NIH 3T3 fibroblasts correlates with the degree of cell spreading^19^. As cells dynamically spread, the initially rounded vertical cross-section of the nucleus becomes progressively flattened^19^. We have reported that fibroblasts on soft substrates spread less and have rounder vertical nuclear cross-sections, while cells on stiff substrates spread more and have flatter nuclear morphologies^20^. In contrast to these observations, we found that individual cells in monolayers had significantly smaller spreading areas than isolated individual cells (Fig. 2A), yet nuclei in monolayers were much flatter than in isolated cells (Fig. 2B). No correlation was found between nuclear aspect ratio and the area of cell spreading in monolayers (Fig. 2C). Rounding caused by depolymerization of F-actin filaments by cytochalasin-D treatment caused substantial nuclear rounding of cells in monolayers (Fig. 2D and Figure S2). Thus, an intact F-actin cytoskeleton that maintains symmetrical cell shapes is required for maintaining flat, uniform cell and nuclear shapes in monolayers.

### E-cadherin adhesion mediated cell shape is required to maintain z-dimensional uniformity in the monolayer

A key difference between monolayers and isolated cells is the presence of E-cadherin mediated cell-cell adhesions that propagate mechanical forces across the monolayer^21^. We therefore disrupted cell-cell adhesions by inhibiting E-cadherin with a function blocking antibody (DECMA) that disrupted cell-cell adhesions^22^. Disruption of cell-cell adhesions caused a loss of flat cell shapes as well as a loss of uniformity of nuclear shapes (Fig. 2D and Figure S2). As E-cadherin adhesions transmit actomyosin pulling forces between neighboring cells^21^,^23^, we next inhibited myosin activity in monolayers with ML-7. ML-7 treatment at a dose of 25 μM for 1 hour is known to inhibit myosin II without disrupting E-cadherin linkages (Figure S2 and S6 and reference^24^). However, ML-7 inhibition had no effect on nuclear shape consistent with similar results in isolated NIH3T3 fibroblasts^19^. The heights of nuclei remained uniform (Fig. 2D,E and Table 1) and flat upon ML-7 treatment (Table 2). Blebbistatin treatment caused curved apical cell shapes and irregular cell-cell contacts (Figure S2, Tables 1 and 2), resulting in increased nuclear heights similar to the results with NIH 3T3 fibroblasts^19^. Similar to our previous study, we conclude here that myosin inhibition affects nuclear shape only when the inhibition affects cell shape.

### Cell morphology determines the vertical shape of the nucleus

**Figure 3:**
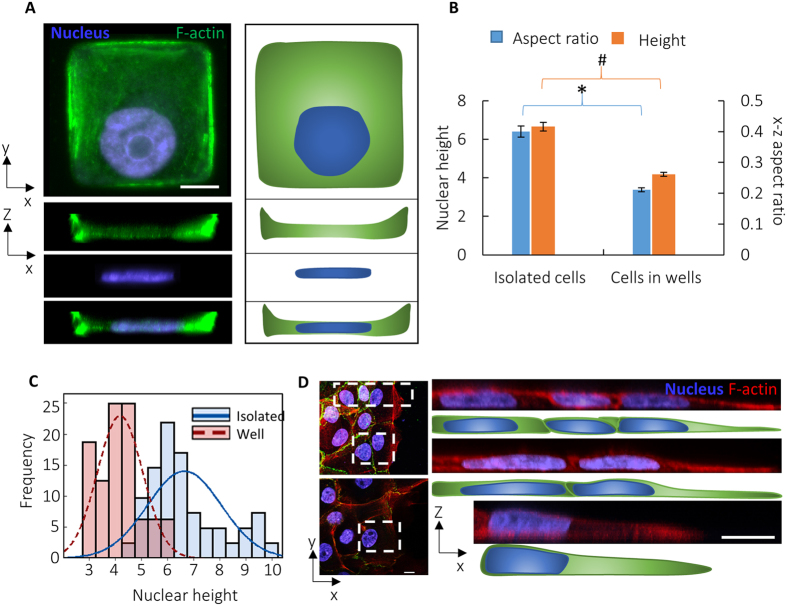
Vertical cell shape determines vertical nuclear shape. (**A**) x-y and corresponding x-z view of a cell cultured in a 5 micron deep square well (left) and corresponding outlines (right). Cells crawl up the sides as seen in the x-z view, resulting in flat x-z cell cross-sections. The nucleus is seen to be correspondingly flat. (**B**) Nuclei in wells are flatter than in isolated cells. *P < 0.05, ^*#*^*P* < 0.05, n = 16 for wells and n >40 for isolated cells. Values are the mean ± SEM. (**C**) The frequency distribution of nuclear heights of isolated cells in wells is narrower than of isolated cells on flat surfaces (see also Table 1), n = 16 for wells and n >40 for isolated cells. (**D**) x-y and corresponding x-z views of cells and nuclei at the edge of a monolayer. Boxed insets are magnified in the x-z view on the right along with sketched outlines. Apical surfaces of cells and nuclei at the edge of the monolayer are curved. Scale bar for all images is 10 μm.

In recent papers, we showed that movements of the nuclear surface are driven by the movements of the proximal cell membrane^25^, and changes in cell shape drive nuclear flattening in NIH 3T3 fibroblasts^19^. We therefore hypothesized that the highly regular and flat nuclear cross-sections are caused by the emergence of regular, flat vertical shapes of cells in the monolayer. To independently create vertical cell shapes that are similar to cell shapes in monolayers, without the presence of cell-cell adhesions, we cultured isolated cells in 5-micron deep square shaped wells and area (two sizes of wells were used: 900 or 1225 μm^2^ in area) similar to the average spreading area of cells in monolayers. This allowed isolated cells to mimic the shapes of cells in a monolayer, but without E-cadherin linkages. The edges of cells in the wells crawled up the sides, ultimately resulting in flat apical cell surfaces (Fig. 3A) unlike the convex apical shapes of isolated cells on flat surfaces. The nuclei of cells in the wells were significantly flatter than isolated cells and also had straighter apical surfaces, similar to those in monolayers (Fig. 3A,B and Table 2). The apical nuclear surface conformed to the flat apical surface of the cell. The variability in nuclear heights in cells in wells was also lower than that in isolated cells on flat surfaces (Fig. 3C and Table 1).

Consistent with the above findings that controlling vertical cell shapes results in uniform and flat nuclear shapes, epithelial cells at the edge of a monolayer had a close concordance between the curvature of the apical cell shape (as cells at the edge do not form cell-cell adhesions on all sides) and the curvature of the nuclear surface (multiple examples are shown in Fig. 3D). Together, these results suggest that it is the cell shape that drives nuclear shaping in epithelial monolayers and uniformity in cell shape is responsible for the high uniformity of nuclear heights.

### Spatial control of the z-dimension

To further understand the mechanical principles by which uniformity is established over long distances in the monolayer, we explored an approach to disrupt uniformity locally in individual cells. We expressed KASH4 to disrupt nuclear cytoskeletal linkages maintained by the LINC complex (linker of nucleoskeleton to cytoskeleton) which transmits mechanical forces between the nucleus and the cytoskeleton^4^,^26^,^27^,^28^,^29^. KASH4 is a domain of nesprin-4, which binds to SUN1/2 in the luminal space between the outer and inner nuclear membranes. Over-expressed GFP-KASH4 competitively inhibits endogenous KASH4 linkages with SUN1/2, thereby disrupting the nucleus-cytoskeletal connections.

**Figure 4:**
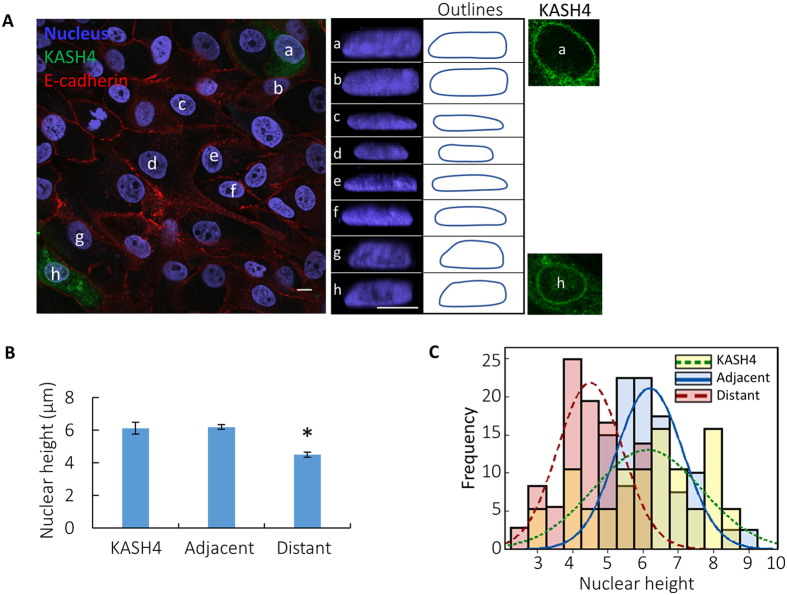
Local disruption of vertical uniformity. (**A**) x-y and x-z view of cells in monolayers transfected with GFP-KASH4. Nucleus ‘a’ and ‘h’ are GFP-KASH4 expressing cells, ‘b’ and ‘g’ are non-transfected cells adjacent to KASH4 expressing cells and, ‘c–f’ are cells farther away from the KASH4 expressing cells(x-y view of the GFP-KASH4 ring and outlines are shown). The nucleus is taller in KASH4 expressing cells and also in immediately adjacent cells, but is flatter farther away. Scale bar is 10 μm for all images. (**B**) Statistical comparison of nuclear heights of KASH4 expressing cells, adjacent cells and cells farther away (distant). *P < 0.01, n >20. Values are the mean ± SEM. (**C**) Frequency distribution of nuclear heights in GFP-KASH4 expressing cells (KASH4), cells adjacent to KASH4 cells (adjacent) and cells farther away from the KASH4 cells (distant). Nuclei in cells adjacent and farther away had significantly lower variance compared to KASH4 cells, n >20.

We located cells expressing GFP-KASH4 that were surrounded by non-transfected cells and imaged nuclear shapes. Nuclear shapes were taller in KASH4 expressing cells than control. Interestingly, nuclei in adjacent, non-transfected cells were also taller, and non-transfected cells farther away had lower heights (Fig. 4A,B). Expression of GFP in individual cells in control experiments demonstrated no such effects (Figure S3). Consistent with these findings, variability in nuclear height was higher in KASH4 expressing cells and non-transfected adjacent cells, but remained unchanged farther away (Table 1 and Fig. 4C). These results establish that LINC complex disruption in one cell in the monolayer influences shapes of nuclei nearby, and the effect on neighboring cells decays with distance.

## Discussion

In this paper, we report that vertical cross-sectional shapes of cells and nuclei in epithelial monolayers are remarkably uniform compared to those in isolated cells. The uniformity in nuclear shapes extends across long distances of several cell lengths. Culturing cells in deep micron sized-wells which enabled cells to climb up the sides resulted in flatter nuclear morphologies of uniform height. This result suggests that it is the cell geometry- specifically the shape of the vertical cross-section of the cell- which establishes the nuclear shape. A similar close concordance between vertical nuclear cross-sections and cell cross-sections is observed in isolated cells on flat surfaces, cells in monolayers, and cells in micron sized-wells. Local disruption of nuclear-cytoskeletal linkages caused a spatial variation of vertical uniformity.

It is likely that cell-cell pulling forces give rise to regular cell cross-sections that result in regular nuclear shapes. How cell shape establishes nuclear shape is less clear. The nuclear surface has been shown to move in response to cell membrane protrusions in NIH 3T3 fibroblasts^25^. It is possible that a similar mechanical principle may apply in monolayers. We found that F-actin disruption with cytochalasin-D treatment disrupted cell-cell adhesions and caused a rounding up of the cell shape (Fig. 2D and Figure S2), and concomitant nuclear rounding. Thus, the mechanical transmission of stress from the cell membrane to the nucleus likely depends on the F-actin cytoskeleton.

Actin stress fibers have been proposed to compress the nucleus and mediate the coordination between cell and nuclear shape in micropatterned cells^30^,^31^. However, myosin inhibition did not affect nuclear heights in the monolayer (Fig. 2D and Table 2). In a previous study, we used laser ablation to sever stress fibers in apposition to the nuclear surface but did not observe an expansion in the nuclear cross-section^25^ in fibroblasts, which argued against a role for compression by contractile stress fibers overlaying the nuclear surface in shaping the nucleus. In contrast, forces on the nuclear surface appear to be tensile in nature in fibroblasts as seen from motion of the nucleus toward newly formed protrusions^32^. Protrusions that occur proximal to the nuclear surface in elongated fibroblasts cause deformation of nuclear shapes without any discernible motion of stress fibers, and nuclear position coincides with the point of maximum tension in the cell in a LINC complex dependent manner^25^. Finally, stress fibers on the apical surface are not visible during initial fibroblast spreading and nuclear flattening^19^. This of course does not argue against latter indentation by stress fibers on the apical surface of the nucleus that has been observed by others^31^. Actomyosin contractile forces can affect nuclear shapes by causing changes in cell shape, as seen in rapid nuclear rounding caused by cell detachment due to trypsinization^19^ or during nuclear shape changes in actomyosin mediated cardiomyocyte beating^33^.

What limits the height of the nucleus? We have previously argued that nuclei are flattened during cell spreading as the nucleus shape changes tends to mimic the changing shape of the cell (horizontal expansion with simultaneous vertical compression); the intervening cytomatrix that links the nuclear membrane to the plasma membrane resists expansion or compression as the cell boundaries move^19^. When an initially round nucleus flattens it must either increase its surface area or reduce its volume (or both). We observed that number of folds or folds in the nuclear lamina decreased with the degree of cell spreading and nuclear flattening of isolated MCF-10A cells (Figure S4). We interpret these folds as excess surface area that allows nuclei to initially flatten during cell spreading without requiring volume^19^. However, once the lamina is unfolded, the nucleus is highly resistant to further area expansion^34^, and further nuclear flattening is hindered as it requires compression of the nuclear volume. Consistent with this interpretation, we found that shRNA knockdown of lamin A/C in MCF-10A cells caused a decrease in nuclear heights and an increase in the degree of cell spreading (Figure S5).

**Figure 5:**
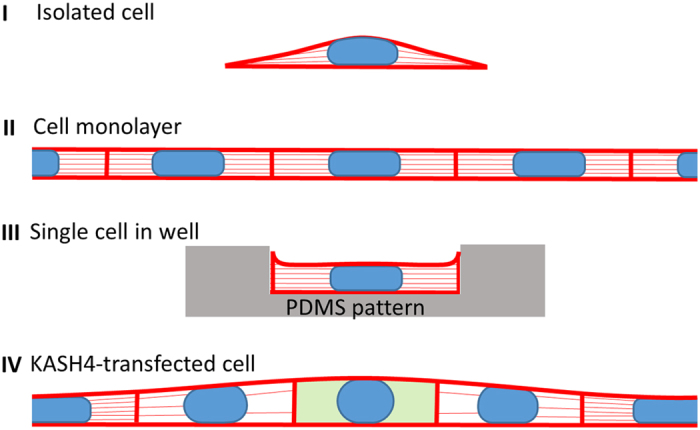
Cartoon depicting the model for nuclear shape in isolated cells and in monolayers. Nuclei are pulled laterally by tensile forces generated during lateral expansion (indicated by ‘tension lines’ in the cell). The simultaneous vertical compression of the cell reduces the nucleus height. Nuclei flatten until the excess lamina is fully unfolded then further flattening is resisted as it requires nuclear volume compression. (**I**) For isolated cells, the amount of tension is higher toward the cell base where lateral expansion is greatest, resulting in a concave apical surface. (**II**) For cells in a monolayer, cell-cell pulling expands cells more uniformly across the vertical dimension, resulting in a more rectangular cross-section, thus a shorter profile for the same nuclear volume. The pulling forces between cells cause the cell heights at the cell-cell junctions to be continuous in both position and slope; this enforces local uniformity in cell heights. (**III**) For a cell in a microfabricated well, the nucleus flattens as the cell spreads laterally then up the walls of the well. The forces on the nucleus and the resulting flat apical surface of the cell and nucleus are similar to those of cells in monolayers. (**IV**) If one cell in the monolayer cannot generate tension on the nucleus, as with KASH4-tranfected cells, the nucleus will resist lateral expansion, resulting in a taller nucleus. The cell-cell pulling forces enforce continuity thereby causing the nearby cells to be similar in height.

A similar interpretation is possible for nuclear flattening in a cell monolayer. In this case, cell-to-cell pulling forces also contribute to pulling and expanding the cells and their nuclei horizontally, but the mechanism and mechanical limitations on nuclear flattening can be similarly explained. Since adjacent cells pull on each other, they tend to form junctions that are continuous in height and slope, as we observed in the monolayer cross-sections (Figs 1F and 3D). Consequently, if one cell is vertically compressed to its minimal height, it will force its neighbors to a similar height. This cell-cell mechanical coupling enforces uniformity in the monolayer (illustrated in Fig. 5). On the other hand, if one cell significantly differs from the other cells in its ability to expand horizontally or compress vertically, it would influence the height of nearby cells. This was observed in the case of the individual KASH4-transfected cells in the monolayer; nuclei of transfected cells were taller, but so were those of nearby untransfected cells. In this case, the lack of nucleus-cytoskeleton LINC-complex connections likely hindered transmission of tensile stress to the nucleus^25^, reducing the lateral expansion of the cell and nucleus and hence the vertical compression of the nucleus (Fig. 5).

With the above physical picture, the reduced nuclear height in the monolayer relative to isolated cells can be simply explained by the geometry. As isolated cells spread, they widen primarily near the cell base and thus should pull horizontally on the nucleus closer to the base, resulting in a curved nuclear apex. In contrast, widening cells in the monolayer should pull on the nucleus more uniformly across the z-direction, resulting in the observed rectangular cross-sections (Fig. 5, I vs II). A nucleus with a rectangular cross-section should have lower height than a nucleus of the same surface area and volume, but with a curved apical surface (see supplementary information for more on this geometrical argument). Nuclei in cells cultured in wells were similarly flat and rectangular in the x-z cross-section as those in monolayers, presumably because they are similarly pulled horizontally, but in this case against the walls of the wells.

In summary, these results support a model in which nuclei in monolayers are pulled into uniform, flat shapes, through a simple mechanism that transmits stresses from the cell membrane to the nuclear surface. This mechanism also effectively explains how nuclear shape mimics cell shape in monolayers. Self-organized vertical uniformity is a characteristic of breast epithelial cell monolayers. Because nuclear shapes can correlate with gene expression in cells^35^,^36^, we speculate that nuclear shape differences may result in differences in gene expression between isolated cells and cells in monolayers.

## Materials and Methods

### Cell culture, transfection and drug treatments

Human breast epithelial cells (MCF 10A from ATCC) were cultured in DMEM/F12 without phenol red supplemented with 5% horse serum, cholera toxin, epidermal growth factor, hydrocortisone, insulin and penicillin streptomycin. Cells were maintained at 37 oC in a humidified, 5% CO_2_ environment. Monolayer culture was performed by seeding cells on fibronectin coated glass-bottomed dishes with 60% confluence and culture for 24 hours. To enrich isolated cells, cells were cultured at 10% confluence. For transfection, the cells were cultured in antibiotic free medium along with the plasmid mixed in Lipofectamine 3000 reagent in OPTI-MEM (Life technologies/Invitrogen). The plasmid EGFP-KASH4 was a kind gift from Kyle Roux. To inhibit E-cadherin linkages cell were treated with anti E-cadherin DECMA antibody (Anti-E-cadherin, clone DECMA-1, rat monoclonal, EMD Millipore) at 50 μg/ml for 4 hours. Myosin activity was inhibited with 25 μM ML-7 treatment for 1 hour or 50 μM Blebbistatin treatment for 1 hour. Actin polymerization was disrupted with 2 μM cytochalasin-D treatment for 1 hour.

### Immunostaining and Imaging

Cells were fixed with 4% paraformaldehyde (Electron Microscopy Sciences, Hatfield, PA) for 10 minutes and washed with PBS before permeabilizing and blocking with permeabilization buffer containing 0.1% triton X-100 in 1% BSA solution for 45 minutes. To stain for E-cadherin, cells were incubated with rabbit monoclonal antibody for E-cadherin (Abcam, Cambridge, MA) at 1:100 dilution for 1 hour in 1% BSA and then incubated with Goat Anti-Rabbit IgG (H + L) antibody (1:200, Life Technologies) at room temperature for 1 hour. To stain for Lamin A/C, the cells were incubated with anti-Lamin A + C antibody (1:500, Abcam, Cambridge, MA) for 1 hour in permeabilization buffer. Cells were then washed and treated with anti-rabbit secondary antibody IgG (H + L) antibody (1:500, Life Technologies) for 1 hour at room temperature. Cells were then stained for F-actin and nucleus with 1:40 Alexa Fluor 488 phalloidin (Invitrogen) and 1:200 Hoechst 33342 (Life Technologies) for 20 minutes at room temperature respectively. Stained cells were imaged with the 60X/1.40 NA oil immersion objective of the Nikon A1 laser scanning inverted confocal microscope (Nikon, Melville, NY).

### Patterning cells in square wells

Silicon wafer masters with square patterns were made with different areas (900 μm^2^, 1225 μm^2^) and 5 μm depth by the photolithography technique. These silicon molds were used to cast a polydimethylsiloxane (PDMS) template by mixing elastomer base/curing agent (Sylgard 184, Dow Corning Corp.) at 10:1 ratio (w/w), degassed and cured at 60 oC for 4 hours^37^. The peeled template had square pillars with 5 μm height. This master PDMS template was cut manually and exposed to a vapor of (tridecafluro-1,1,2,2-tetrahrdrooctyl)-1-tricholorosilane (T2492-KG, United Chemical Technologies) in vacuum for 45 minutes before pouring PDMS mixture to avoid formation of covalent bonds between the PDMS template and the PDMS^38^. This was again degassed, cured at 60 oC for 4 hours and allowed to dry before peeling the negative stamp that had the square shaped wells with 5 μm height. This stamp was then plasma treated for 30 seconds and coated with 50 μg/ml fibronectin solution, incubated in vacuum chamber for 5 minutes (to get rid of bubbles trapped in the wells) and then for 1 hour at room temperature before seeding the cells at low confluence. After 24 hours of cell culture, the cells were fixed, stained, and the PDMS stamp was inverted and mounted on a glass bottom dish (such that the side of the PDMS with cells faces the glass bottom) with ProLong Gold Antifade Mountant (Life Technologies) for imaging.

## Additional Information

**How to cite this article**: Neelam, S. *et al*. Vertical uniformity of cells and nuclei in epithelial monolayers. *Sci. Rep.* **6**, 19689; doi: 10.1038/srep19689 (2016).

## Supplementary Material

Supplementary Information

## Figures and Tables

**Table 1:** Statistical comparison of variances with the F-test.

MCF10 A	n	Nucleus Height (μm)	Standard deviation	Comparison of variance
Monolayer (control)	63	4.1	0.8	–
Isolated cells	42	6.8	1.5	Higher
Cells in wells	16	4.2	0.9	Equal
Monolayer (Untreated)	63	4.1	0.8	–
E-cad inhibited	46	5.5	1.1	Higher
DMSO	38	4.5	0.9	–
Cytochalasin-D	58	5.9	1.2	Higher
ML-7	61	4.6	0.9	Equal
Blebbistatin	55	6.0	1.2	Higher
GFP-KASH4 (control)	20	6.12	1.5	–
Adjacent	43	6.2	0.9	Equal
Distant	36	4.5	0.9	Lower

For the F-test, all comparisons are with respect to corresponding controls in the sub-table. ‘n’ refers to the number of cells.

**Table 2:** Nuclear shape and cell spreading.

MCF10 A	n	Nucleus Height	Nucleus Major axis	Nucleus Aspect ratio	Cell spreading area
Monolayer(untreated) glass	63	4.1 ± 0.1	18.9 ± 0.3	0.22 ± 0.01	1151 ± 59
Monolayer on soft substrate	37	6.8 ± 0.2	17.3 ± 0.4	0.4 ± 0.02	801 ± 60
Isolated cells	42	6.7 ± 0.2^	17.0 ± 0.4^	0.40 ± 0.02^	1697 ± 93^
Cells in wells	15	4.2 ± 0.2^X^	20.0 ± 0.5 ^X^	0.2 ± 0.01 ^X^	1085 ± 33^X^
Treatments					
E-cad inhibited	46	5.5 ± 0.2*	18.0 ± 0.3	0.31 ± 0.01*	1535 ± 89*
DMSO	38	4.5 ± 0.1	19.3 ± 0.3	0.23 ± 0.01*	1285 ± 49*
ML-7	61	4.5 ± 0.1	18.6 ± 0.3	0.24 ± 0.01	908 ± 51*
Cytochalasin-D	58	5.9 ± 0.2^#^	16.4 ± 0.2	0.37 ± 0.01^#^	892 ± 40^#^
Blebbistatin	55	6.0 ± 0.2^#^	17.5 ± 0.2	0.34 ± 0.01^#^	1126 ± 82^#^

Nuclear heights, Major axis in x-y view, aspect ratio (height/major axis), and cell spreading area were measured across different conditions. Statistically significant differences are indicated by symbols. ^P < 0.05 relative to monolayer, ^X^P < 0.05 relative to isolated cells, *P < 0.05 relative to monolayer, ^#^P < 0.01 relative to DMSO.
