# Supplementary material for: Vertical uniformity of cells and nuclei in epithelial monolayers
Source: Sci Rep. 2016 Jan 22;6:19689. doi: 10.1038/srep19689 (PMC4726213; doi:10.1038/srep19689)

Supplementary information

Vertical uniformity of cells and nuclei in epithelial monolayers

Srujana Neelam[b], Peter Hayes[a], Qiao Zhang[a], Richard B. Dickinson[a] and Tanmay P. Lele[a, *]

Figure S1. Histogram plots compare the frequency distribution of aspect ratios in x-y view of cells in monolayers and isolated cells. Variance in x-y aspect ratio is not different.

Figure S2. x-y and corresponding x-z views of cells of monolayers in control, cytochalasin-D treated, E-cadherin inhibited, ML-7 treated and blebbistatin treated conditions. Treatment with cytochalasin-D, blebbistatin and E-cadherin antibody disrupted the cell-cell linkages and caused rounded nuclear apexes. Statistical data is available in Tables 1 and 2 for these conditions.

Figure S3. Flat nucleus in GFP control cells. (A) x-y and x-z view of cells in monolayers transfected with GFP. Nucleus 'a' is GFP expressing cell, 'b' is non-transfected cell adjacent to GFP expressing cell and, 'c' is cell farther away from the GFP expressing cell. The corresponding x-z views of the nuclei show no effect of GFP expression. (B) The plot compares the nuclear heights of cells expressing GFP and GFP-KASH4. The height of the nucleus in GFP-KASH4 cells is significantly higher. $*P < 0.05$, $n = 20$. Values are the mean ± SEM.

Figure S4. (A) x-y view of isolated MCF 10A cells seeded and fixed at 30 mins, 60 mins, 6 hours and 24 hours. The nuclei are stained with lamin A/C (green) and show clear folds/ wrinkles at earlier time points and at 24 hours the surface of the nucleus is completely smooth. (B) The plot shows the ratio of nuclei with grooves to the total number of nuclei at each time point. As the seeding time increased, less nuclei with grooves were found. N=3 replicates from n> 120 nuclei per time point. (C) The plot shows the nuclear height at each time point. The nuclei are flat after 6 hours of seeding. N=3 replicates from n> 48 nuclei per time point. (B) and (C) Values are the mean ± SEM. $*P < 0.05$, comparing to 24 hours of seeding.

Figure S5. (A) MCF 10A cells stably expressing scrambled shRNA (control) and shLMNA were cultured in monolayers. Top panel show the x-y view of nuclei immunostained for lamin A/C and the bottom panel shows the monolayers immunostained for E-cadherin and nucleus. (B) x-z aspect ratio and nuclear height of the cells expressing shLMNA are significantly smaller than the scrambled. $*P < 0.05$, $n > 30$, $^{\#}P < 0.05$, $n > 30$ for the indicated comparisons. Values are the mean ± SEM. (C) Plot shows that the cell spreading area of cells stably expressing shLMNA is significantly larger.

Figure S6. Validation of ML-7 treatment. Similar to the results of (Shewan et al., 2005), phosphomyosin staining was observed in cell-cell contacts in control monolayers, while this staining was lost upon ML-7 treatment. Plot shows quantitative comparisons of averaged corrected total cell fluorescence using the method of (McCloy et al., 2014); images were captured under constant microscope settings for control and ML-7 treatment. * corresponds to $p<0.01$.

Geometrical explanation for decreased height of the nucleus in a monolayer compared to isolated cells.

Here we show that a nucleus with a flat apical surface will have a smaller height H than one with a rounded apical surface, for the same surface area, A, and volume V. We represent the nucleus as a short cylinder of radius a and height b, with a spherical cap of height h, as shown in the cross-section below. Since A and V are assumed fixed, the dimensions a and b depend on h.

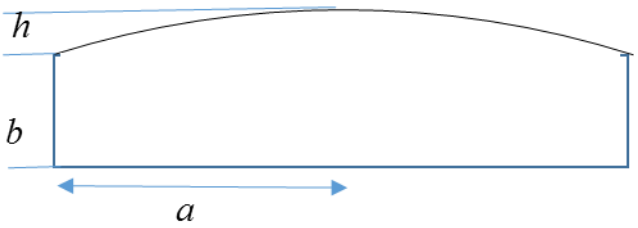

The total height of the nucleus is $H = b + h$. When curved cap region increases in height, some of nuclear volume is contained in the cap, and the cylindrical volume and height must reduce correspondingly. The total height increases with *h* when

$$\frac{dH}{dh} = b' + 1 > 0 \qquad (1)$$

where $b' \equiv \frac{db}{dh}$ The total area and volume are

$$A = \pi(a^2 + h^2) + \pi a^2 + 2\pi ab \qquad (2)$$

$$V = \frac{\pi}{6}(3a^2 h + h^3) + \pi a^2 b, \qquad (3)$$

respectively. We look at the case where the apical surface is nearly flat and ask whether the inequality in Eq. 1 holds. Setting the derivatives of constant *A* and *V* with respect to zero,

$$\left.\frac{dA}{dh}\right|_{h=0} = 4\pi a a' + 2\pi(ab' + a'b) = 0 \qquad (4)$$

$$\left.\frac{dV}{dh}\right|_{h=0} = \frac{\pi}{2}a^2 + 2\pi a a' b + \pi a b' = 0 \qquad (5)$$

yields *a'* and *b'* at h = 0,

$$a' = \frac{a}{2(2a-b)} \qquad b' = -\frac{(2a+b)}{2(2a-b)} \qquad (6)$$

Plugging *b'* into the inequality Eq 1, provides the condition where the total height increases with *h*: $a > \frac{3}{2}b$

Hence, for a nucleus with a radius greater than 1.5X its height (typically the case for cells), it is expected to have a lower height when the apical nuclear surface is flat in a monolayer (*h* = 0) rather than rounded (in isolated cells).

REFERENCES

McCloy, R.A., Rogers, S., Caldon, C.E., Lorca, T., Castro, A., and Burgess, A. (2014). Partial inhibition of Cdk1 in G 2 phase overrides the SAC and decouples mitotic events. Cell Cycle *13*, 1400-1412.

Shewan, A.M., Maddugoda, M., Kraemer, A., Stehbens, S.J., Verma, S., Kovacs, E.M., and Yap, A.S. (2005). Myosin 2 is a key Rho kinase target necessary for the local concentration of E-cadherin at cell-cell contacts. Mol Biol Cell *16*, 4531-4542.

Figure S1

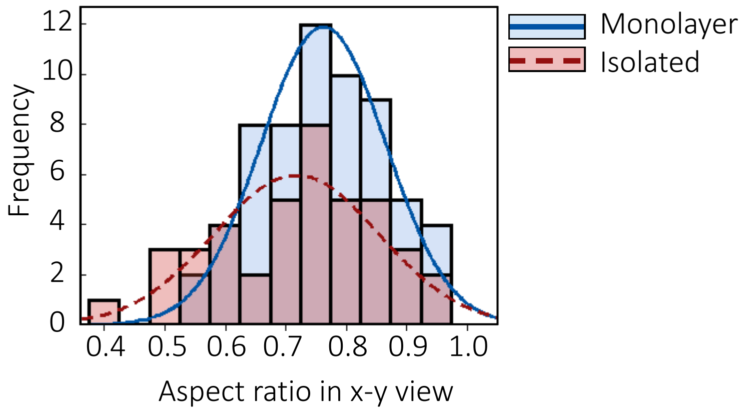
Monolayer
Isolated
Frequency
12
10
8
6
4
2
0
0.4
0.5
0.6
0.7
0.8
0.9
1.0
Aspect ratio in x-y view

Figure S2

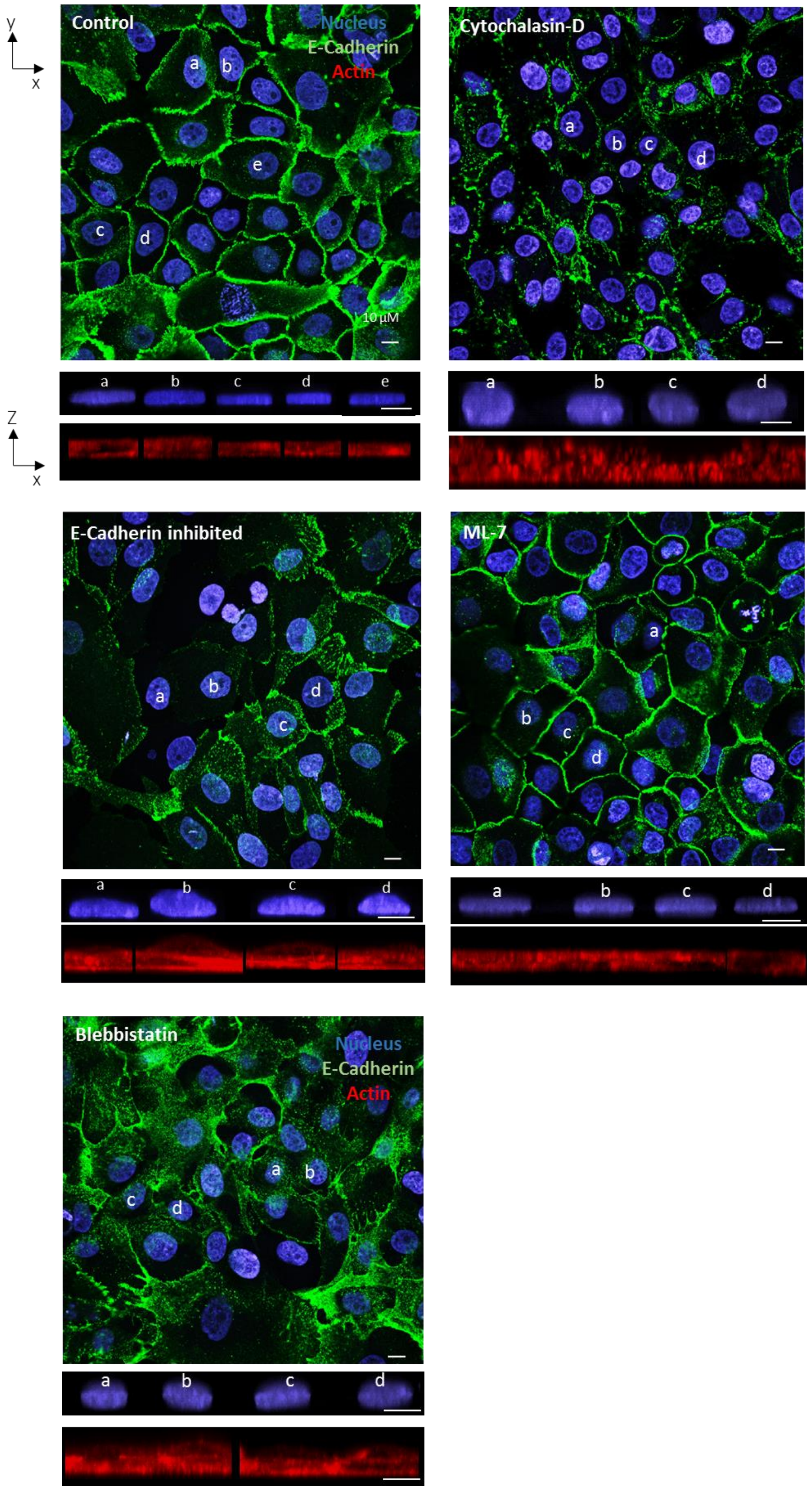

Control
Nucleus
E-Cadherin
Actin
10 µM
Cytochalasin-D
E-Cadherin inhibited
ML-7
Blebbistatin
Nucleus
E-Cadherin
Actin

Figure S3

A

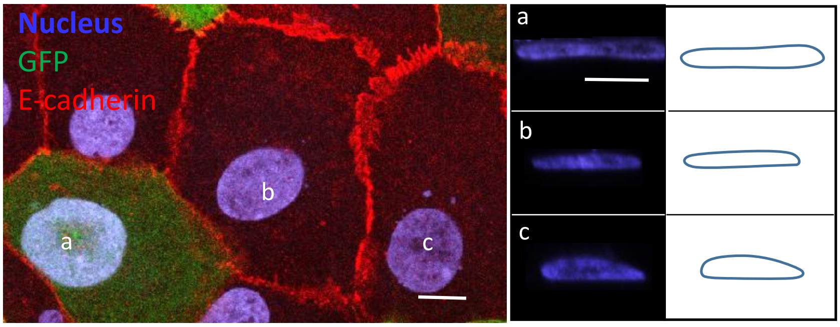

Nucleus
GFP
E-cadherin
a
b
c
a
b
c

B

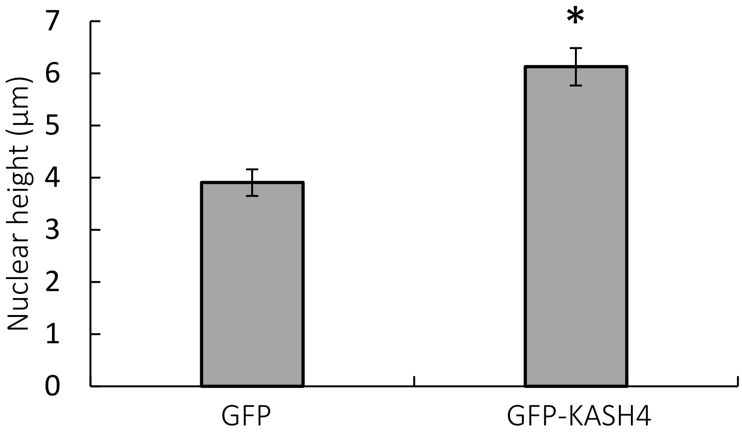

7
6
5
4
3
2
1
0
Nuclear height (µm)
*
GFP
GFP-KASH4

Figure S4

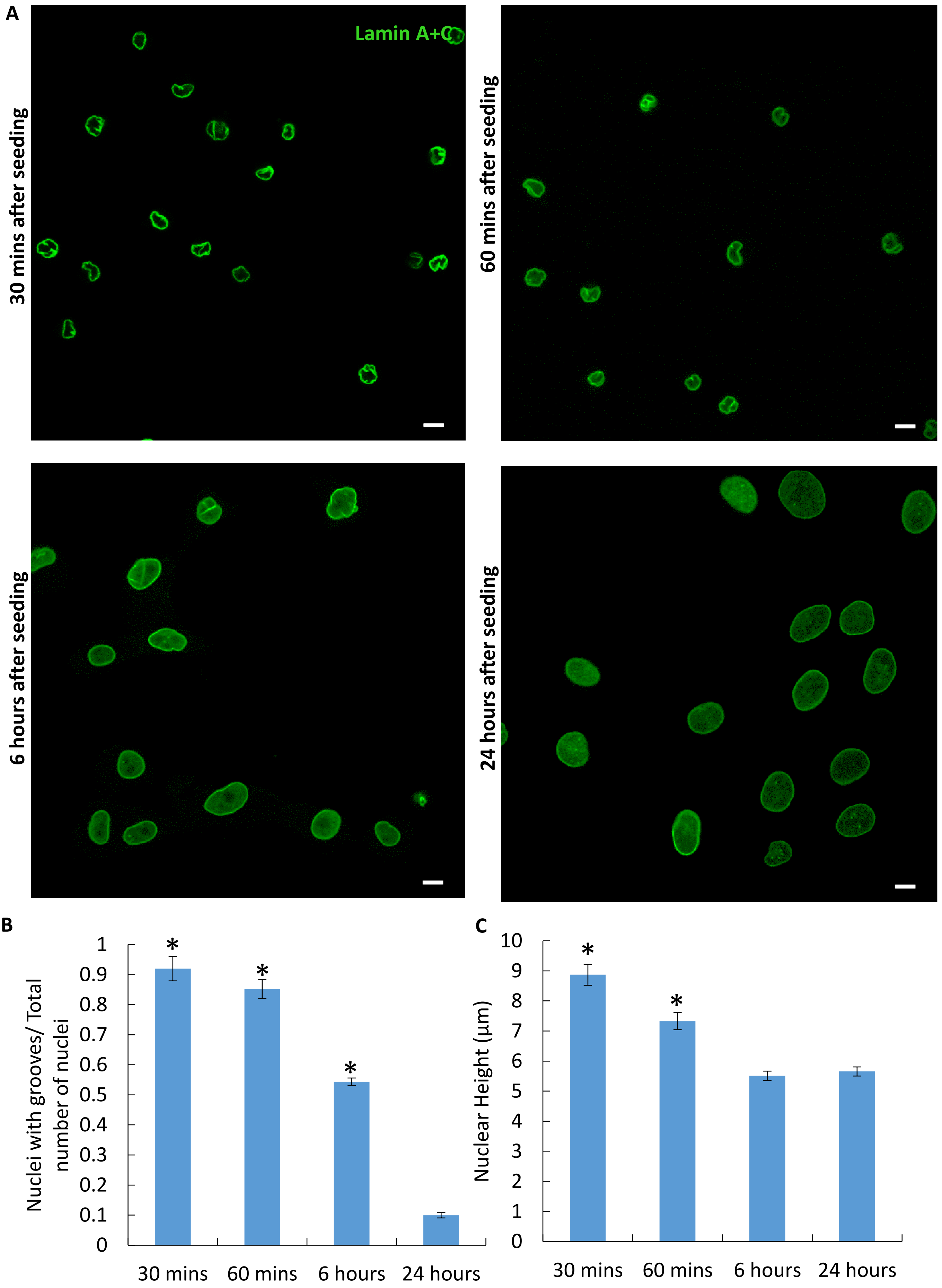
A
Lamin A+C
30 mins after seeding
60 mins after seeding
6 hours after seeding
24 hours after seeding
B
Nuclei with grooves/ Total number of nuclei
1
0.9
0.8
0.7
0.6
0.5
0.4
0.3
0.2
0.1
0
*
30 mins
60 mins
6 hours
24 hours
C
Nuclear Height (µm)
10
9
8
7
6
5
4
3
2
1
0
30 mins
60 mins
6 hours
24 hours

Figure S5

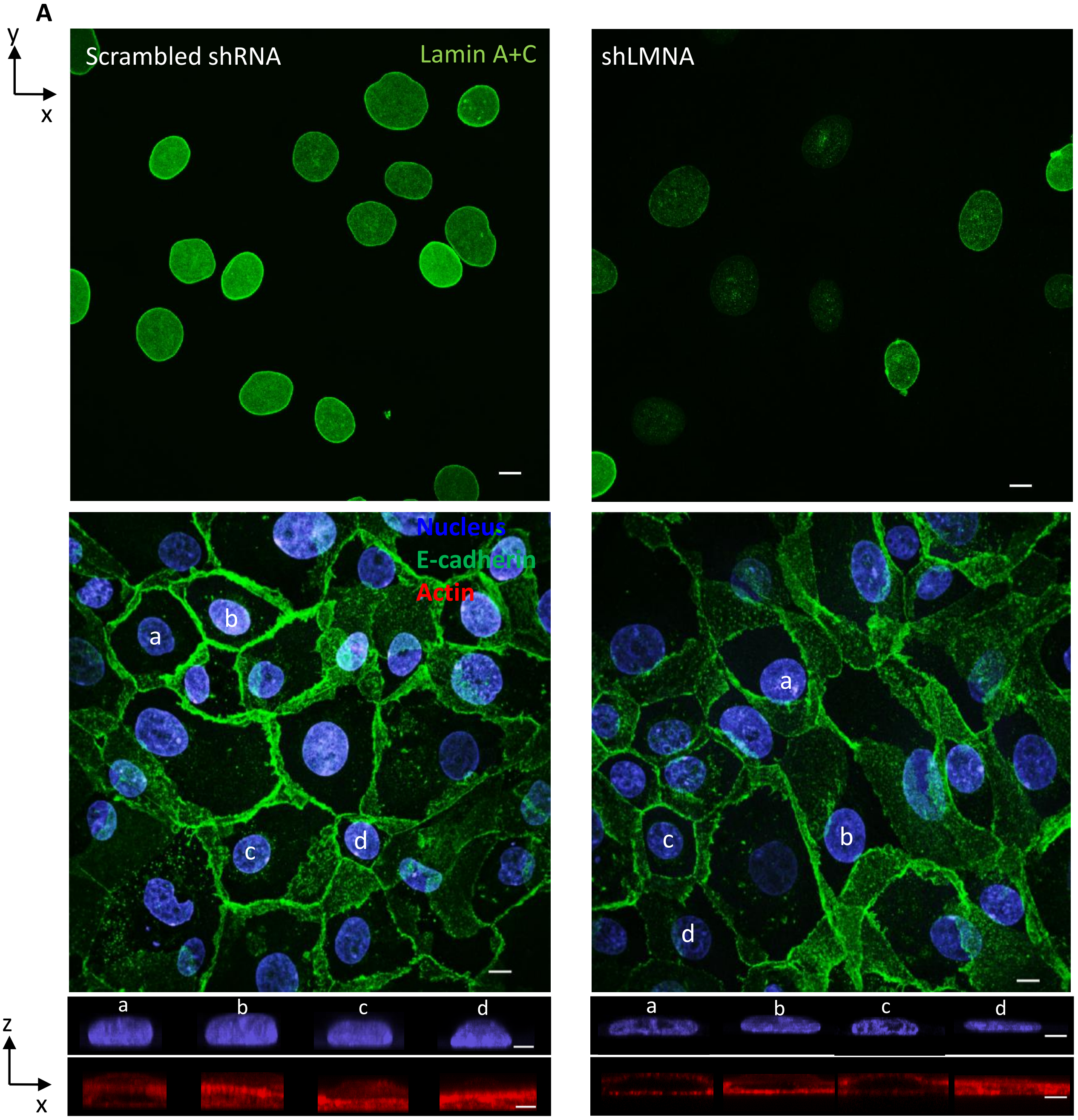

A
y
x
Scrambled shRNA
Lamin A+C
shLMNA
Nucleus
E-cadherin
Actin
a
b
c
d
z
x

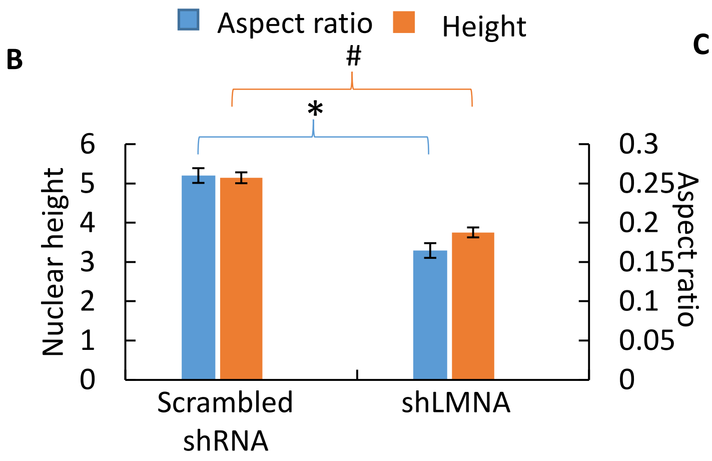

B
Aspect ratio
Height
#
*
Nuclear height
Aspect ratio
6
5
4
3
2
1
0
0.3
0.25
0.2
0.15
0.1
0.05
0
Scrambled shRNA
shLMNA

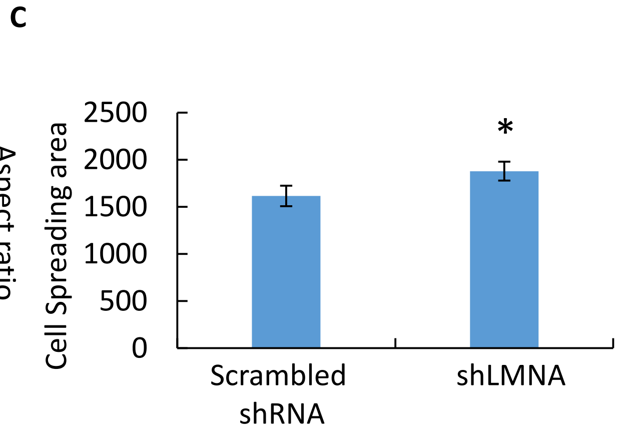

C
Cell Spreading area
2500
2000
1500
1000
500
0
*
Scrambled shRNA
shLMNA

Figure S6

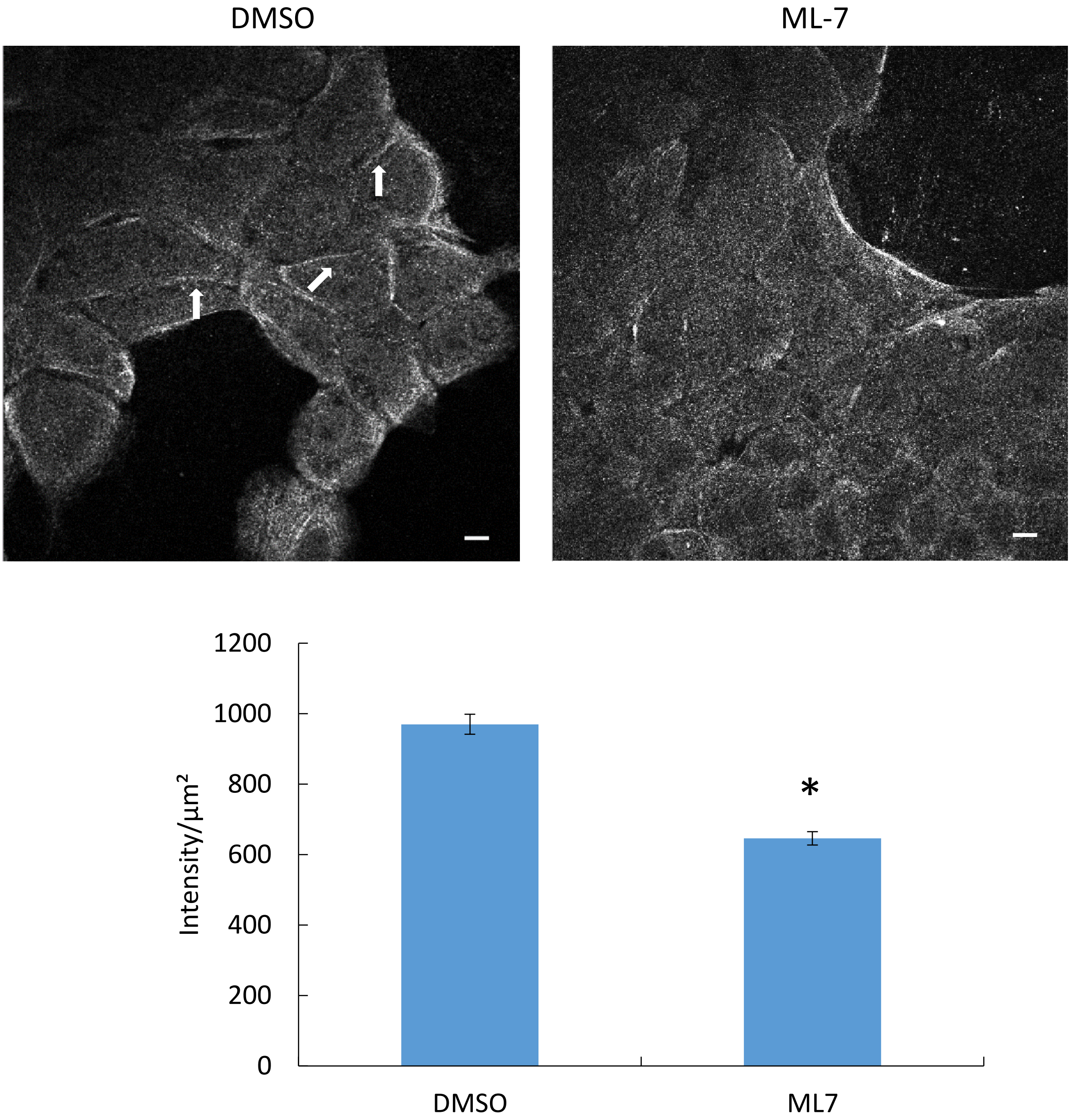

Supplement: Supplementary Information [file srep19689-s1.pdf]
